# Transcriptome analysis reveals candidate genes involved in quercetin biosynthesis in *Euphorbia maculata*

**DOI:** 10.1038/s41598-025-00794-w

**Published:** 2025-05-17

**Authors:** Sanbao Guo, Meiling Song, Mingming Gui, Qingyang Wu, Wuhua Yu, Chunxiang Chen, Zechang Rao, Shenghe Huang

**Affiliations:** 1 Department of Pharmacy, Jiangxi College of Traditional Chinese Medicine, Fuzhou, 344000 China; 2 Department of Basic Medicine, Jiangxi College of Traditional Chinese Medicine, Fuzhou, 344000 China; 3 Fuzhou Medical College, Nanchang University, Fuzhou, 344000 China

**Keywords:** *Euphorbia maculata*, Transcriptome, Quercetin, Gene annotation, Expression characteristics, Genetics, Molecular biology, Plant sciences

## Abstract

An investigation was conducted through transcriptome sequencing in various tissues at different stages to explore the quercetin biosynthesis pathway in *Euphorbia maculata*. A total of 83,028 unigenes was assembled utilizing Trinity software, with an N50 length of 1721 bp and a mean length of 1004 bp. Among these unigenes, 51,822 were annotated in six public databases. The transcriptome analysis revealed 45,727 CDS sequences and 56 TF families. Candidate genes involved in quercetin biosynthesis were also revealed, including phenylalanine ammonia-lyase (17 unigenes), cinnamate 4-hydroxylase (3 unigenes), 4-coumarate-CoA ligase (16 unigenes), chalcone synthase (5 unigenes), chalcone isomerase (4 unigenes), flavanone 3-hydroxylase (1 unigene), flavonoid 3′-hydroxylase (4 unigenes), and flavonol synthase (9 unigenes). Additionally, 42 key differentially expressed genes (DEGs) related to quercetin biosynthesis were identified in the same tissues at different stages, with 35 DEGs exhibiting down-regulated expression and 7 DEGs displaying up-regulated expression. These findings not only enhance the genetic knowledge of *E. maculata*, but also establish a basis for further investigating the mechanism of quercetin biosynthesis, and improving the quality of *E. maculata*.

## Introduction

*E. maculata*, a herbaceous plant belonging to the *Euphorbiaceae* family and *Euphorbia* genus, is indigenous to the eastern regions of North America and is commonly observed in agricultural lands. Now, it is widely distributed in China, with the exception of the Qinghai-Tibet Plateau, and is prevalent across all geographical areas^1^. *E. maculata* is known for its medicinal properties, which include attributes such as ‘cooling blood and stopping bleeding’, ‘clearing heat and detoxification’, and ‘eliminating dampness and jaundice’. It is commonly used to treat dysentery, hematuria, hematochezia, jaundice, and carbuncle toxin^2^. The chemical components of *E. maculata* are complex and mainly consist of flavonoids, tannins, coumarins, and organic acids. Active monomer components such as quercetin, rutin, kaempferol, myricetin, and gallic acid have been identified in *E. maculata*^3,4^. Currently, the research on the molecular biology of *E. maculata* is rare, and its genetic information is relatively lacking. This limitation hinders the development of basic research on this plant.

**Fig. 1:**
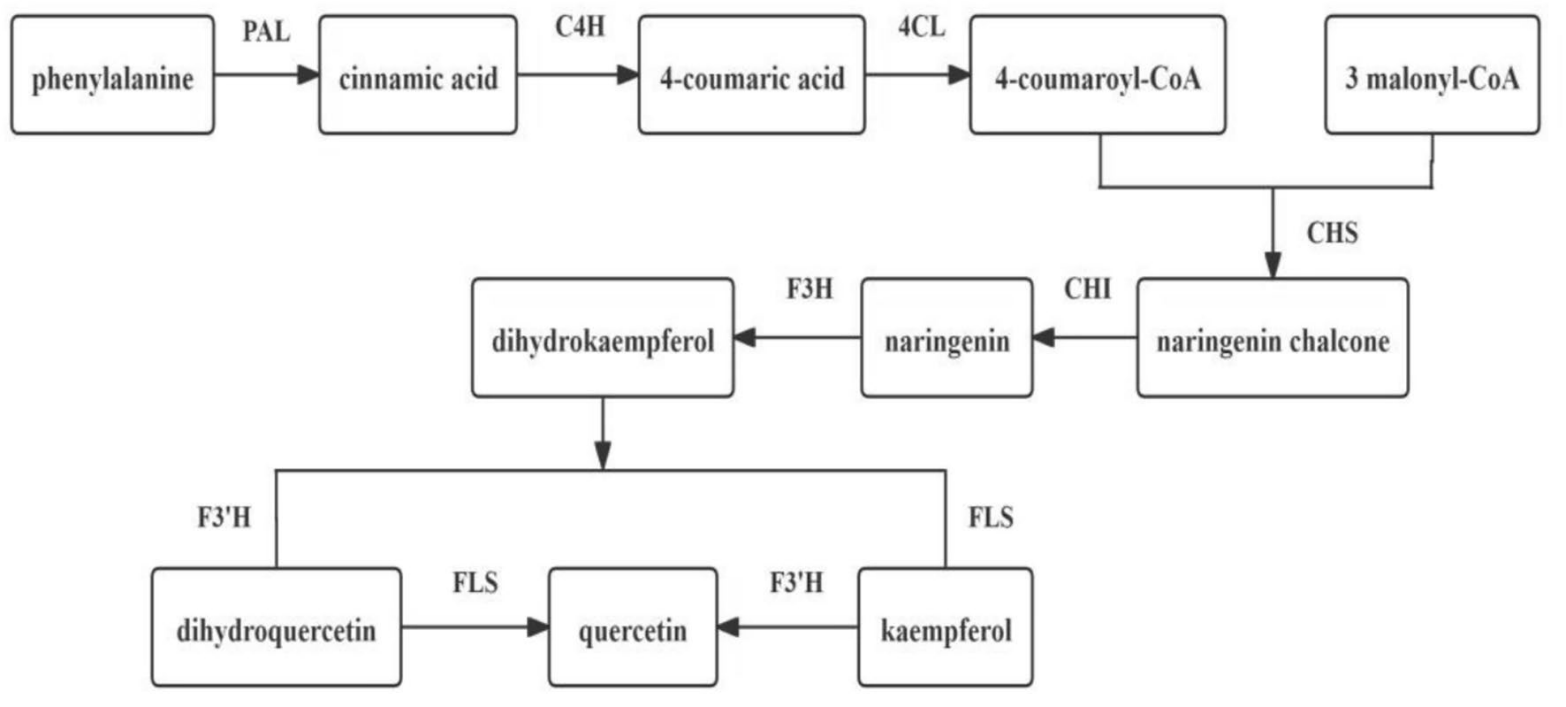
Biosynthesis pathway of quercetin in plants. (PAL) Phenylalanine ammonia-lyase. (C4H) Cinnamate 4-hydroxylase. (4CL) 4-coumarate-CoA ligase. (CHS) Chalcone synthase. (CHI) Chalcone isomerase. (F3H) Flavanone 3-hydroxylase. (F3′H) Flavonoid 3′-hydroxylase. (FLS) Flavonol synthetase.

Transcriptome sequencing is a widely used method for studying gene expression regulation. Several studies have utilized this technique to identify candidate genes involved in flavonoid biosynthesis. For example, in the transcriptome analysis of *Ziziphora bungeana*, 60 unigenes were found to play a role in flavonoid biosynthesis, encoding 13 key enzymes^5^. Similarly, in the transcriptome data of *Stellaria yunnanensis* root, 80 unigenes were identified to be involved in flavonoid biosynthesis, encoding 16 key enzymes^6^. In the transcriptome analysis of *Sophora japonica*, 218 unigenes were discovered to be associated with rutin biosynthesis, encoding 8 key enzymes^7^. Flavonoids are important secondary metabolites in plants and serve as one of the primary active ingredients in traditional Chinese medicine. Quercetin, a flavonol compound, is synthesized through the phenylpropanoid metabolic pathway. This process involves the catalysis of phenylalanine by phenylalanine ammonia-lyase (PAL), cinnamate 4-hydroxylase (C4H), and 4-coumarate-CoA ligase (4CL) to produce 4-coumaryl-CoA. Subsequently, chalcone synthase (CHS) catalyzes one 4-coumaroyl-CoA and three malonyl-CoA to produce naringenin chalcone, which serves as the starting material for flavonoid biosynthesis. Finally, naringenin chalcone is converted to quercetin through the catalysis of chalcone isomerase (CHI), flavanone 3-hydroxylase (F3H), flavonoid 3′-hydroxylase (F3′H), and flavonol synthase (FLS)^8–10^ (Fig. 1). Modern pharmacological studies have shown that quercetin possesses antiviral, antibacterial, antioxidant, hepatoprotective, and antitumor properties, making it one of the main active components of *E. maculata*^11,12^. It should be noted that quercetin is the only indicator component used to determine the content of *E. maculata* in the Chinese Pharmacopoeia, which specifies that the dried product of *E. maculata* should contain at least 0.1% quercetin^2^. However, there have been no reports on the quercetin biosynthesis pathway in *E. maculata*. Therefore, obtaining genomic information of *E. maculata* through transcriptome sequencing is crucial in elucidating the mechanism of quercetin biosynthesis, which has significant implications for the quality formation of *E. maculata*.

The active ingredients of most medicinal plants are secondary metabolites, and their content differs during the plants’ developmental stages and in varying tissues^13^. Previous research has indicated variances in the levels of quercetin in various tissues of *E. maculata* at different growth phases, with the highest levels in the leaves during the vegetative stage and the lowest in the roots during the reproductive stage^14^. However, the underlying molecular mechanisms responsible for the variations in quercetin content remain undisclosed, primarily due to limited genomic data available for *E. maculata*. Therefore, to explore the molecular mechanism of quercetin biosynthesis in *E. maculata*, its biosynthesis pathway was analyzed for the first time by transcriptome data. Based on the KEGG annotation information, candidate genes involved in quercetin biosynthesis were identified. This study provides insights into the molecular mechanism of quercetin biosynthesis in *E. maculata* and offers a significant genetic resource for developing varieties with improved quality using genetic engineering.

## Materials and methods

### Plant materials

*E. maculata* samples were gathered from the experimental field of Jiangxi College of Traditional Chinese Medicine (Fuzhou, China) at the vegetative stage (pre-flowering with a minimum of 2 above-ground branches) and reproductive stage (with at least 3 fruits). The plant materials were identified by Associate Professor Canhui Tang. At the vegetative stage, *E. maculata* was categorized into root (VPR), stem (VPS), and leaf (VPL), while at the reproductive stage, it was divided into root (RPR), stem (RPS), leaf (RPL), and fruit (RPF). Each experimental sample consisted of a mixture of 3 or more plants, which were immediately frozen in liquid nitrogen and stored at − 80 °C. Three independent replicates were collected for each sample.

### Transcriptome sequencing

Total RNA from each sample was extracted using the Ultrature RNA Kit (Cowin Biotech, Taizhou, China). The quality and quantity of the total RNA were assessed using agarose gel electrophoresis, Nanodrop 2000 (Thermo Fisher Scientific, Waltham, USA), and Agilent 2100 (Agilent Technologies, Santa Clara, USA). The construction and normalization of cDNA libraries were carried out using the Hieff NGS^®^ Ultima Dual-mode mRNA Library Prep Kit (Yeasen, Shanghai, China). The library quality was assessed using Agilent 2100, and qualified libraries were subjected to transcriptome sequencing on Illumina NovaSeq 6000 (Illumina, San Diego, USA).

### Sequence assembly and functional annotation

Raw reads were filtered to generate clean reads by removing reads containing adapter, reads with N ratio greater than 10%, reads with all A bases, and low-quality reads. Then, high-quality clean reads were assembled into contigs using Trinity software^15^. The longest contig for all genes were extracted from the assembled contigs. These sequences were clustered to identify the unigenes using CD-HIT-EST v4.8.1^16^. These unigenes were functionally annotated by aligning them against the Nr^17^, SwissProt^18^, KEGG^19,20^, KOG^21^, and PFAM^22^ databases using Blast, and unannotated unigenes were mapped onto the published *Euphorbia* genomes such as *Euphorbia lathyris* and *Euphorbia peplus*, with an E-value cut-off of 1 × 10^–5^. Unigenes sequence of *E. maculata* were aligned against the Nr database, and the sequence with the best alignment result (the lowest E-value) of each unigene in the Nr database was taken as the corresponding homologous sequence (if there was a juxtaposition which the first was taken) to determine the species of the homologous sequence. The number of homologous sequences of each species was counted and used as a criterion for determining the genetic relationship between *E. maculata* and other species. GO^23^ functional annotation was performed using the Blast2GO program^24^. The transcription factors were identified using hmmscan alignment of the sequences against the Plant TFdb database^25^.

### CDS analysis

According to the established priority order, the unigenes of *E. maculata* were aligned using Blast against the Nr, SwissProt, KEGG, and KOG database to determine the coding sequence. Subsequently, the CDS sequence was then translated into the corresponding amino acid sequence. Unigenes that were not aligned were analyzed using TransDecoder^26^ software (https://github.com/TransDecoder/TransDecoder/wiki) to identify the coding sequence and translate it into the amino acid sequence.

### Differential expression analysis

To compare the gene expression across different stages in the root, stem, and leaf of *E. maculata*. Gene expression level was estimated by RSEM^27^ for each sample: clean reads were mapped back onto the assembled transcripts and read counts of each gene was obtained from the mapping results. The reads per kilobase of transcript per million mapped reads (FPKM) method was performed to calculate the normalized gene expression levels. Differentially expressed genes (DEGs) analysis of two groups was performed using the DESeq2^28^. DESeq2 provides statistical procedures to determine differential expression in digital gene expression data using a model based on the negative binomial distribution. The resulting P-values were adjusted using the Benjamini–Hochberg method for controlling the false discovery rate (FDR). DEGs were screened with the threshold of false discovery rate (FDR) < 0.05 and the absolute value of fold change (FC) > 2. Additionally, KEGG pathway enrichment analysis was performed on the DEGs.

## Results

### Transcriptome sequencing and sequence assembly

The tissue samples from *E. maculata* were collected at the vegetative stages (root, stem, and leaf) and reproductive stages (root, stem, leaf, and fruit) for transcriptome sequencing. After rigorous filtering and quality assessment of the raw reads, a total of 53,946,749, 42,462,786, and 46,280,825 clean reads were obtained in the root, stem, and leaf at the vegetative stages, respectively. Similarly, 45,262,520, 48,790,599, 41,905,421, and 41,850,502 clean reads were obtained in the root, stem, leaf, and fruit at the reproductive stages, respectively.

**Fig. 2:**
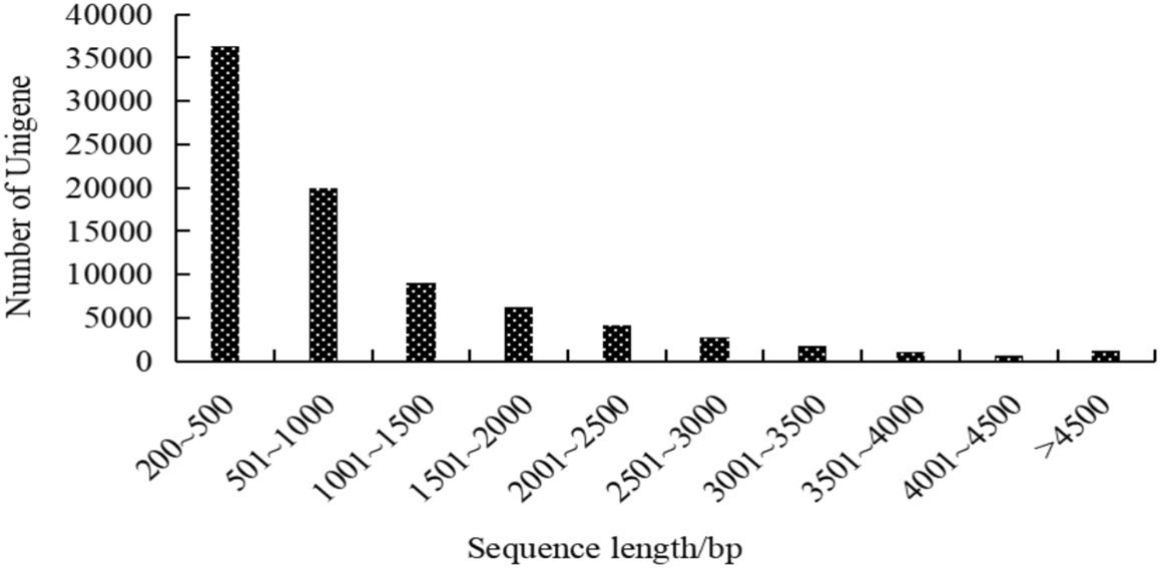
Distribution of length of unigenes from *E. maculata.*

The clean reads were *de nove* assembled into contigs using Trinity software, resulting in a total of 83,028 unigenes with an N50 length of 1721 bp, N90 length of 408 bp, and mean length of 1004 bp (Table 1). All unigenes were longer than 200 bp, with 56,230 unigenes (67.72%) long from 200 to 1000 bp, and 11,518 unigenes (13.87%) were longer than 2000 bp (Fig. 2).

**Table 1:** Transcriptome assembly quality statistics of *E. maculata.*

Number of genes	GC percentage (%)	N50 (bp)	N90 (bp)	Max length (bp)	Min length (bp)	Average length (bp)	Total assembled bases
83,028	40.80	1721	408	15,569	201	1004	83,397,325

### Functional annotation

For functional annotation in public databases, the unigenes were aligned using BlastX against the Nr, GO, KOG, KEGG, Swissprot, and PFAM databases. The majority of unigenes (57.12%) were annotated in the Nr database, while the fewest number of annotated unigenes (32.38%) were annotated in the KOG database. A total of 51,822 unigenes (62.42%) were annotated in at least one database, and 31,206 unigenes (37.58%) were not annotated (Table 2). Of the 31,206 unannotated unigenes, 587 (1.88%) and 433 (1.39%) unigenes were annotated in the genomes of *E. peplus*^29^ and *E. lathyris*^30^, respectively.

**Table 2:** Statistics of the annotation for the assembled unigenes in public databases.

Annotation database	Number of unigenes	Percentage (%)
Nr	47,427	57.12
GO	36,438	43.89
KOG	26,881	32.38
KEGG	44,879	54.05
SwissProt	33,760	40.66
PFAM	32,406	39.03
Annotation	51,822	62.42
Without annotation genes	31,206	37.58

**Fig. 3:**
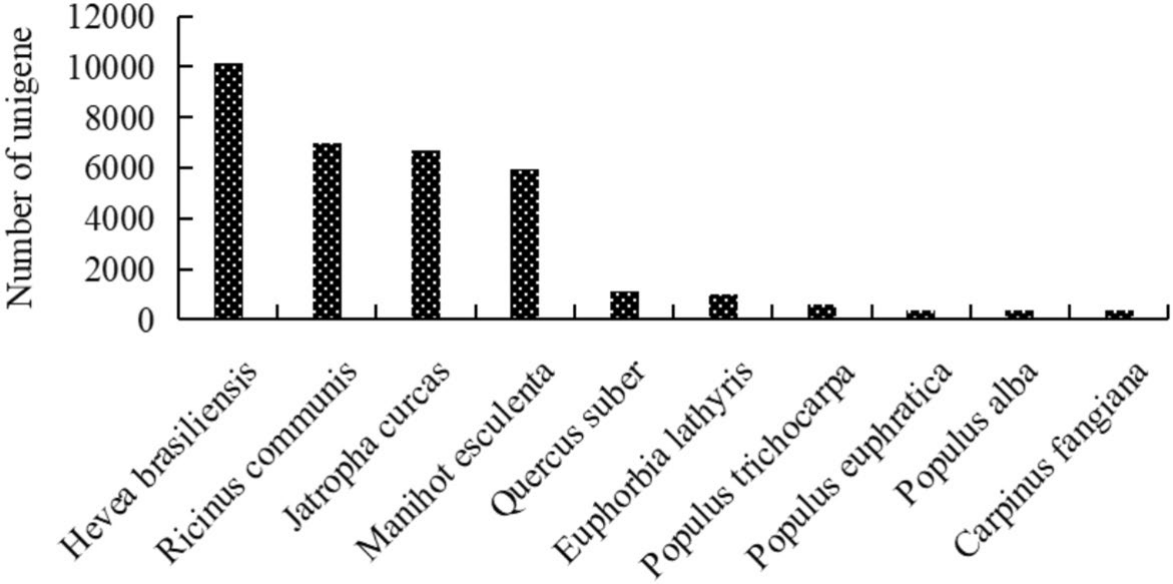
Major species distribution of unigenes annotated in the Nr database.

In the Nr database, comparative analysis of *E. maculata* unigenes with other species revealed ten species with close genetic relationships. Notably, *Hevea brasiliensis* showed the closest genetic relationship to *E. maculata*, with 10,146 similar sequences (21.39%), followed by *Ricinus communis* (6965 unigenes, 14.69%), *Jatropha curcas* (6677 unigenes, 14.08%), *Manihot esculenta* (5956 unigenes, 12.56%), and *Quercus suber* (1108 unigenes, 2.34%) (Fig. 3).

**Fig. 4:**
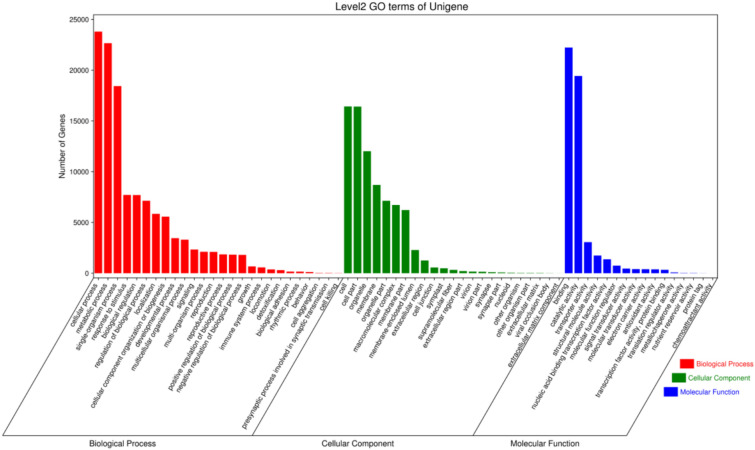
GO classification of transcriptomic unigenes.

A total of 36,438 unigenes were categorized into three GO categories (Molecular function, Cellular component, and Biological process) and 65 subcategories based on sequence homology. The predominant subcategories within each major category were ‘Cellular process’ (23,798 unigenes), ‘Cell & Cell part’ (16,428 unigenes), and ‘Binding’ (22,228 unigenes). By contrast, a minority of unigenes fell under ‘Cell killing’ (20 unigenes), ‘Extracellular matrix component’ (5 unigenes), and ‘Chemoattractant activity’ (1 unigenes) (Fig. 4).

**Fig. 5:**
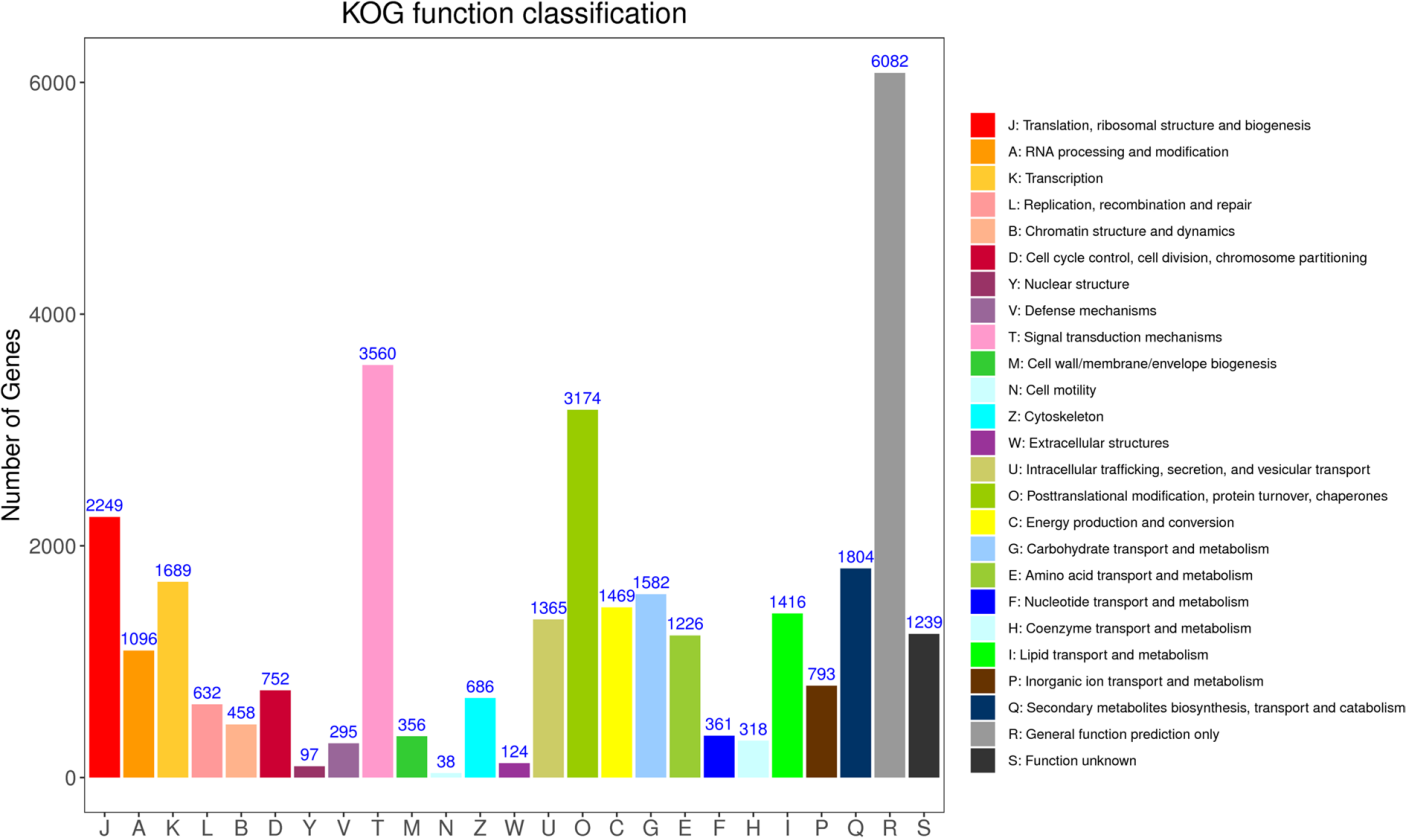
KOG function classification of transcriptomic unigenes.

KOG analysis showed a total of 26,881 unigenes clustered into 25 functional categories based on their significant hits. The most representative category was ‘General function prediction only’, encompassing 6082 unigenes, constituting 22.63% of all unigenes. Substantial proportions of unigenes were also classified into ‘Signal transduction mechanisms’, ‘Posttranslational modification, protein turnover, chaperones’, ‘Translation, ribosomal structure and biogenesis’ and ‘Secondary metabolite biosynthesis, transport and catabolism’, with 3560 (13.24%), 3174 (11.81%), 2249 (8.37%), and 1804 (6.71%) unigenes, respectively. By contrast, quite a few unigenes were annotated in ‘Cell motility’ with 38 unigenes (0.14%). Furthermore, 1239 unigenes (4.61%) with unknown functions necessitate further exploration (Fig. 5).

**Fig. 6:**
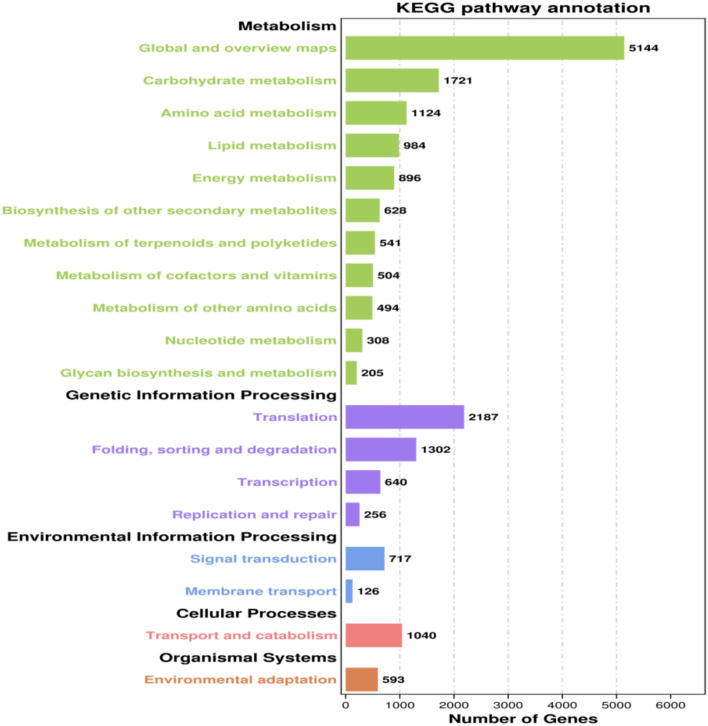
KEGG functional classification and pathway assignment of transcriptomic unigenes.

Unigenes were searched against the KEGG database to unveil the biological pathways of *E. maculata*. Overall, 11,291 unigenes were mapped to 138 KEGG pathways and classified into 5 functional groups. The most prominent pathways were ‘Metabolism’ (12,549 unigenes), followed by ‘Genetic information processing’ (4385 unigenes), ‘Cellular processes’ (1040 unigenes), ‘Environmental information processing’ (843 unigenes), and ‘Organismal systems’ (593 unigenes) (Fig. 6). The main medicinal ingredients present in herbal plants include phenylpropanoids, flavonoids, alkaloids, terpenes, steroids, and glycosides. In total, 17 KEGG pathways were involved in the biosynthesis of these medicinal ingredients in *E. maculata*. The investigation focused on the flavonoid biosynthesis pathway, revealing that the most significantly enriched pathway was ‘Phenylpropanoid biosynthesis’ (392 unigenes), followed by ‘Flavonoid biosynthesis’ (142 unigenes), ‘Anthocyanin biosynthesis’ (23 unigenes), ‘Isoflavonoid biosynthesis’ (13 unigenes), ‘Flavone and flavonol biosynthesis’ (12 unigenes), and ‘Betalain biosynthesis’ (12 unigenes) (Table 3).

**Table 3:** Biosynthesis pathway of the medicinal ingredients in *E. maculata.*

Metabolic pathway	Number of unigenes	Pathway ID	Medicinal ingredients
Phenylpropanoid biosynthesis	392	ko00940	Phenylpropanoids
Flavonoid biosynthesis	142	ko00941	Flavonoids
Isoflavonoid biosynthesis	13	ko00943	
Flavone and flavonol biosynthesis	12	ko00944	
Anthocyanin biosynthesis	23	ko00942	
Betalain biosynthesis	12	ko00965	
Isoquinoline alkaloid biosynthesis	43	ko00950	Alkaloids
Caffeine metabolism	6	ko00232	
Indole alkaloid biosynthesis	8	ko00901	
Tropane, piperidine and pyridine alkaloid biosynthesis	51	ko00960	
Monoterpenoid biosynthesis	68	ko00902	Terpenes
Diterpenoid biosynthesis	36	ko00904	
Sesquiterpenoid and triterpenoid biosynthesis	111	ko00909	
Terpenoid backbone biosynthesis	141	ko00900	
Brassinosteroid biosynthesis	43	ko00905	Steroids
Steroid biosynthesis	95	ko00100	
Glucosinolate biosynthesis	22	ko00966	Glycoside

### Transcription factors identification

**Fig. 7:**
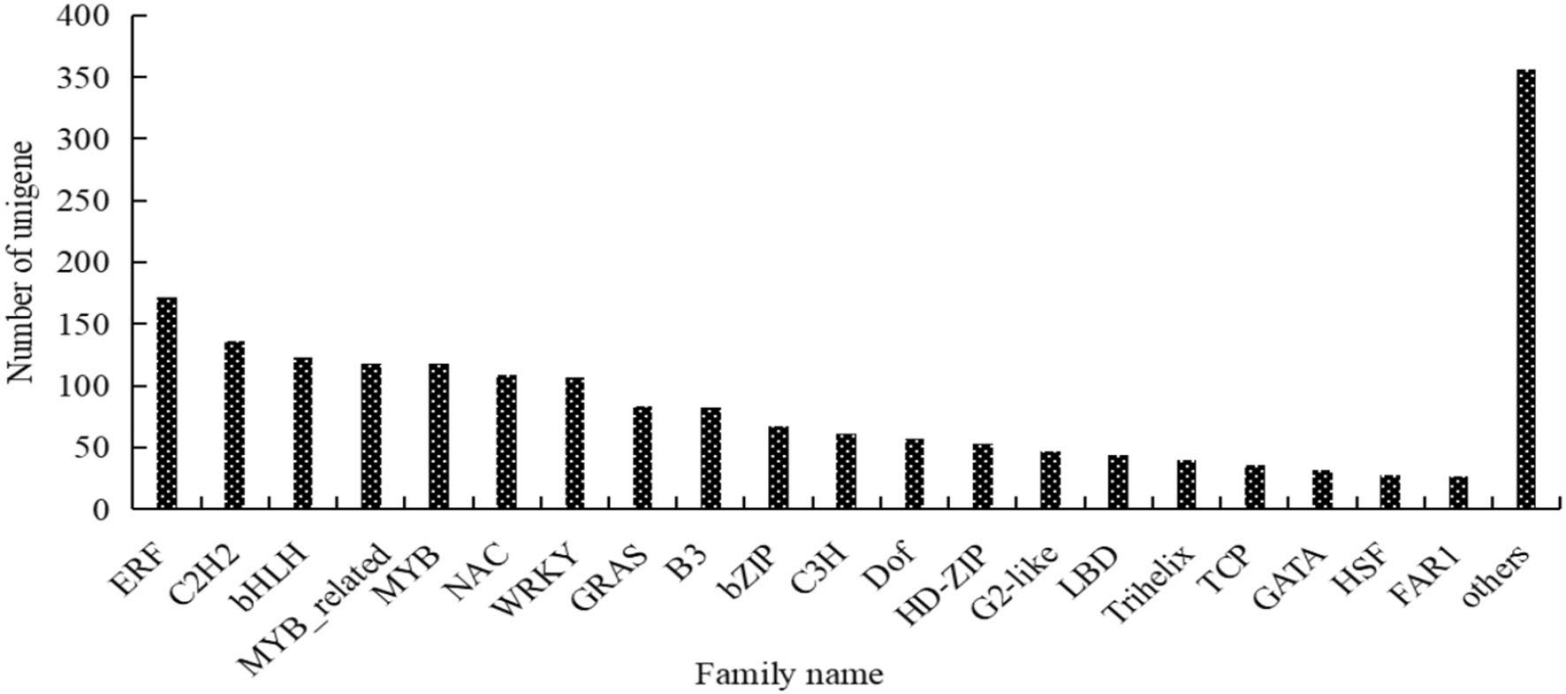
Distribution of transcription factors in *E. maculata.*

The transcription factors (TFs) were identified using hmmscan. A total of 1896 unigenes encode potential TFs, which can be sorted into 56 TF families. The ERF transcription factor (172, 9.07%) was the largest family, followed by C2H2 (136, 7.17%), bHLH (123, 6.49%), MYB-related (118, 6.22%), and MYB (118, 6.22%). Additionally, 356 unigenes (18.78%) were classified into other 36 transcription factor families (Fig. 7).

### Candidate genes related to quercetin biosynthesis

Quercetin, the primary indicator component of *E. maculata* in Chinese Pharmacopoeia (2020 edition), was studied to explore the mechanisms underlying its biosynthesis. Through KEGG annotation information, a set of 59 candidate genes associated with quercetin biosynthesis were identified, including 17 *PAL*, 3 *C4H*, 16 *4CL*, 5 *CHS*, 4 *CHI*, 1 *F3H*, 4* F3*′*H*, and 9 *FLS* (Table 4).

**Table 4:** Candidate genes related to the quercetin biosynthesis pathway.

Nos	Name	Abbreviation	KO ID	Number of unigenes
1	Phenylalanine ammonia-lyase	PAL	K10775	17
2	Cinnamate 4-hydroxylase	C4H	K00487	3
3	4-coumarate-CoA ligase	4CL	K01904	16
4	Chalcone synthase	CHS	K00660	5
5	Chalcone flavanone isomerase	CHI	K01859	4
6	Flavanoid 3′-hydroxylase	F3H	K00475	1
7	Flavanone 3-hydroxylase	F3′H	K05280	4
8	Flavonol synthase	FLS	K05278	9

### CDS sequences identification

**Fig. 8:**
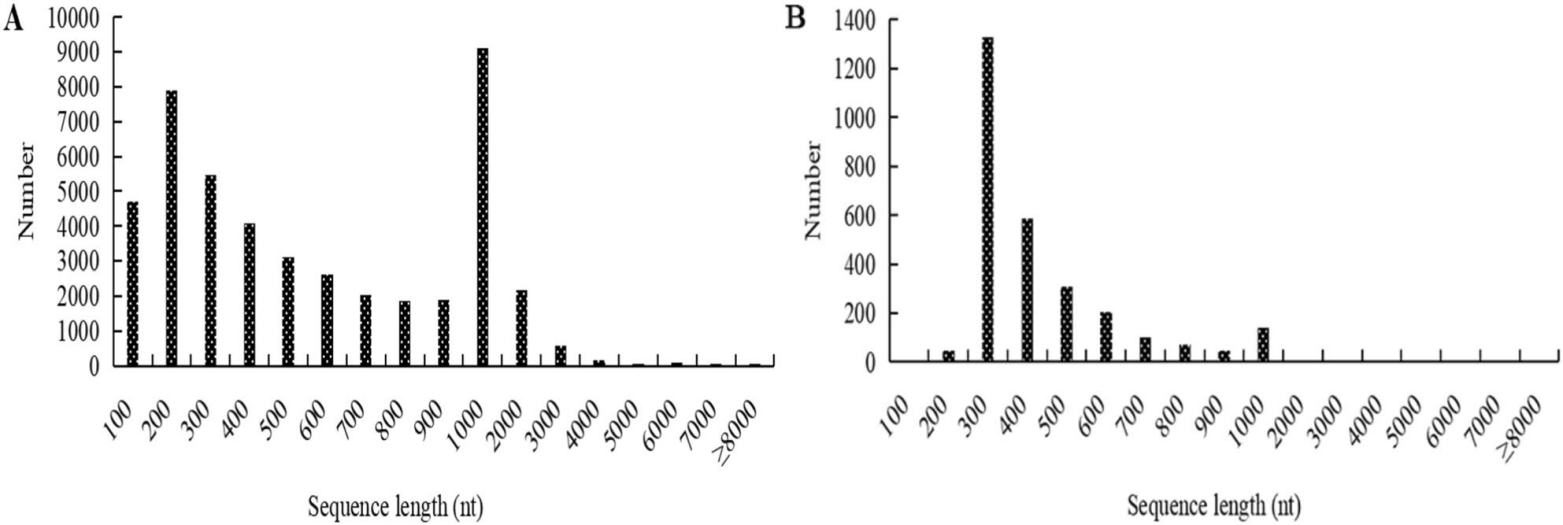
CDS sequence length and distribution. (**A**) CDS sequence length and distribution using Blast prediction. (**B**) CDS sequence length and distribution using TransDecoder prediction.

Unigenes were aligned against databases like Nr, SwissProt, KEGG, and KOG, leading to the identification of 45,727 CDS sequences through Blast analysis. The majority of these sequences fell within the length range of 100 to 2000 nt (44,870, 98.13%), with 17,028 CDS sequences (37.24%) being long between 700 and 2000 nt (Fig. 8A). The unaligned unigenes were further predicted using TransDecoder, resulting in the discovery of 2840 CDS sequences. These sequences were primarily between 300 and 600 nt in length (2431 sequences, 85.60%), with 360 CDS sequences (12.68%) being long between 700 and 2000 nt (Fig. 8B).

### Differential gene expression analysis in the same tissues at different stages

**Fig. 9:**
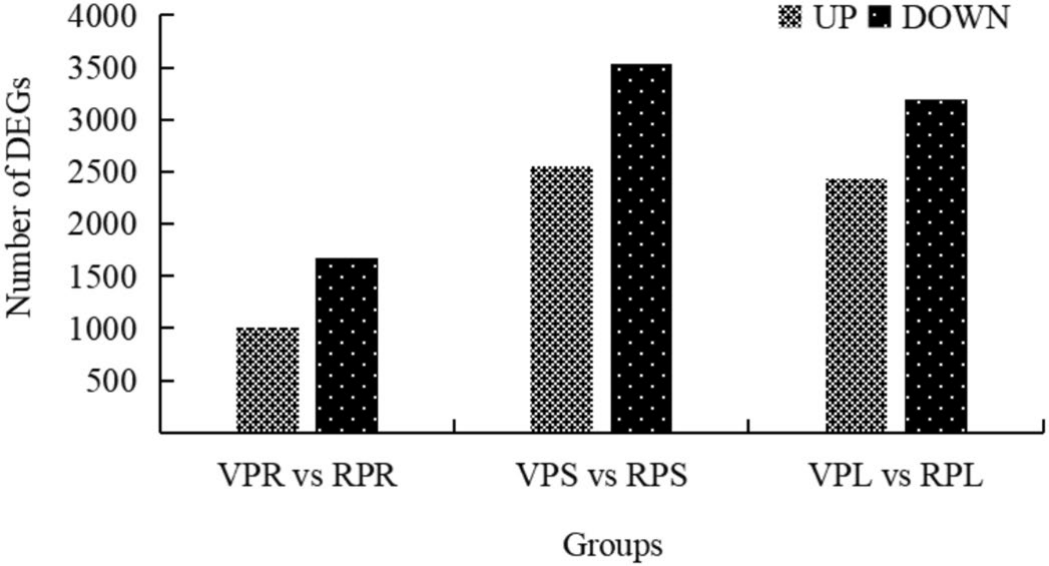
The number of up-regulated and down-regulated DEGs in three tissues of *E. maculata*. Root at vegetative stage (VPR). Stem at vegetative stage (VPS). Leaf at vegetative stage (VPL). Root at reproduction stage (RPR). Stem at reproduction stage (RPS). Leaf at reproduction stage (RPL).

The analysis of differentially expressed genes (DEGs) revealed significant changes in gene expression patterns between vegetative and reproductive stages in the root, stem, and leaf tissues of *E. maculata*. Compared to the same tissues at the vegetative stage, 2676, 6078, and 5631 DEGs were identified in the root (with 1002 genes up-regulated and 1674 genes down-regulated), stem (with 2546 genes up-regulated and 3532 genes down-regulated), and leaf (with 2435 genes up-regulated and 3196 genes down-regulated) at the reproductive stage, respectively (Fig. 9).

To elucidate the biological pathways of the DEGs, KEGG pathway analysis was performed. Among 2676 DEGs of the ‘VPR vs RPR’ comparison, 560 DEGs were mapped to 117 KEGG pathways. In the ‘VPS versus RPS’ comparison, 951 DEGs were mapped to 127 KEGG pathways, and in the ‘VPL vs RPL’ comparison, 1058 DEGs were mapped to 121 KEGG pathways. Further analysis of the KEGG pathway related to quercetin biosynthesis in the root, stem, and leaf, there were 64, 76, and 69 genes involved in ‘Phenylpropanoid biosynthesis’, 14, 29, and 19 genes were involved in ‘Flavonoid biosynthesis’, and 1, 3, and 0 genes were involved in ‘Flavone and flavonol biosynthesis’ (Table 5). These results show that as *E. maculata* matured, the number of down-regulated genes related to the quercetin biosynthesis pathway was more than up-regulated genes in the same tissue. Previous study has indicated that quercetin content in the same tissue showed a downward trend as *E. maculata* matured^14^, suggesting the decrease of quercetin accumulation may be related to these down-regulated genes.

**Table 5:** The number of DEGs involved in the quercetin biosynthesis pathway.

Metabolic pathway	Pathway ID	Number of up/down-regulated genes		
		VPR versus RPR	VPS versus RPS	VPL versus RPL
Phenylpropanoid biosynthesis	ko00940	2/62	32/44	12/57
Flavonoid biosynthesis	ko00941	0/14	15/14	7/12
Flavone and flavonol biosynthesis	ko00944	0/1	1/2	0/0

To understand the regulation mechanisms of quercetin biosynthesis in *E. maculata*, key DEGs were identified in the same tissues at different stages. Compared to the same tissues at the vegetative stage, there were 8 *PAL*, 4 *4CL*, 1 *CHS*, and 3 *FLS* in the root, 3 *PAL*, 3 *4CL*, 1 *CHS*, 1 *CHI*, and 2 *FLS* in the stem, and 9 *PAL*, 3 *4CL*, 1 *CHS*, and 3 *FLS* in the leaf at the reproductive stage, respectively. Overall, in the same tissues, *PAL*, *4CL*, *CHS*, *CHI*, and *FLS* showed significantly lower expression at the reproductive stage than at the vegetative stage (Table 6). This suggests that quercetin biosynthesis in *E. maculata* may be significantly correlated with these key DEGs.

**Table 6:** The number of candidate genes related to quercetin biosynthesis DEGs.

Nos.	Abbreviation name	Number of up-regulated/down-regulated genes		
		VPR versus RPR	VPS versus RPS	VPL versus RPL
1	PAL	0/8	2/1	3/6
2	4CL	1/3	1/2	0/3
3	CHS	0/1	0/1	0/1
4	CHI	0/0	0/1	0/0
5	FLS	0/3	0/2	0/3

## Discussion

*E. maculata* is a plant of significant medicinal value in traditional Chinese, Mongolian, and Uyghur medicine. However, the lack of genomic and transcriptomic data has posed a major obstacle to fundamental research on this plant species. To fill this gap, transcriptome sequencing was conducted across various tissues of *E. maculata* at different developmental stages, resulting in the identification of 83,028 unigenes with an N50 length of 1721 bp and a mean length of 1004 bp. These results demonstrate the high quality of the assembled sequence (N50 > 800 bp)^6^ and the abundance of genetic information available for *E. maculata*, both of which are essential for transcriptome analysis. These findings provide valuable genetic resources for further research on the biosynthesis pathway of secondary metabolites and biodiversity in *E. maculata* as well as other related species.

Bioinformatics tools were employed to analyze the unigenes of *E. maculata*. A total of 51,822 (62.42%) unigenes were annotated, significantly lower than the annotation rates of *Ampelopsis grossedentata* (91.07%)^31^, *Elsholtzia bodinieri* (89.68%)^32^, and *Ziziphora bungeana* (72.87%)^5^, indicating a substantial proportion of unigenes with undefined functions and sequence characteristics. Notably, the annotation rate of 62.42% is higher than that of other *Euphorbia* plants, such as *Euphorbia fscheriana* (42.7%), *Euphorbia ehracteolata* (44.38%)^33^, *Euphorbia tirucalli* (51.08%)^34^ and *Euphorbia kansui* (62.36%)^35^. This may be due to the presence of new genes in *E. maculata*, some unigenes having shorter fragment lengths, limited genomic studies of related species, and the lack of genome and protein sequence information for *Euphorbia* in public databases. 31,206 unannotated unigenes were mapped to the genomes of *Euphorbia peplus* and *Euphorbia lathyris*, and the results showed that the annotation rates were relatively low for both. This may results from the large number of *Euphorbia* species (about 2000) and the large differences between species.

Unigenes of *E. maculata* were analyzed for the involvement in secondary metabolites biosynthesis based on the KEGG annotation information. Phenylpropanoids, flavonoids, alkaloids, terpenes, steroids, and glycosides are the primary medicinal ingredients found in herbal plants^7^. A total of 17 KEGG pathways were revealed as being involved in the biosynthesis of these six ingredients in *E. maculata*. Quercetin is a flavonol compound and this study mainly focused on the quercetin biosynthesis pathways, including ‘Phenylpropanoid biosynthesis’, ‘Flavonoid biosynthesis’ and ‘Flavone and flavonol biosynthesis’. There were 392, 142, and 12 genes involved in these three pathways, respectively. The quercetin biosynthesis pathway and its key enzymes have been clarified^8–10^. In this study, we revealed 59 candidate genes for quercetin biosynthesis from *E. maculata*. These findings provide the foundation for future research on cloning, identification, and regulation of key genes involved in quercetin biosynthesis.

Extensive research has been performed on the flavonoid biosynthesis pathway in plants, and it is widely accepted that genes related to this pathway can be classified into two main categories: structural genes and regulatory genes^36^. Transcription factors play a crucial role in regulating plant metabolism by initiating transcription programs for genes which either inhibit or promote the synthesis of secondary metabolites. The regulatory genes involved in flavonoid biosynthesis include MYB-bHLH-WDR, MYB, WRKY, and ERF TFs etc.^37,38^. MYB has been found to have a significant impact on flavonol biosynthesis^39^. MYB11, MYB12, and MYB111 belong to SG7 of the R2R3-MYB family. These proteins controlled flavonol biosynthesis by independently activating expression of *CHS*, *CHI*, *F3H*, and *FLS1* in *Arabidopsis thaliana*^38^. In tomato, overexpression of *CsERF003* from citrus promoted expression of *PAL*, *C4H*, *4CL*, *CHS*, *CHI*, *F3H*, and *FLS* to increase accumulation of flavonol glycosides and naringenin chalcone^40^. In grape, overexpression of *VqWRKY31* from *Vitis quinquangularis* activated expression of *CHS*, *CHI*, *FHT*, *FLS*, and* F3*′*5*′*H* to increase flavonoid content^41^. *OsbZIP48* was identified as a positive regulatory gene of flavonoid biosynthesis in rice^42^. The genome of *E. maculata* has been found to contain 56 transcription factor families, with 172 ERF (9.07%), 118 MYB (6.22%), 107 WRKY (5.64%), and 67 bZIP (3.53%). Candidate genes involved in quercetin biosynthesis include *PAL*, *C4H*, *4CL*, *CHS*, *CHI*, *F3H*,* F3*′*H*, and *FLS*. However, the role of ERF, MYB, bZIP, and WRKY TFs in regulating key quercetin synthase genes have not been reported in *E. maculata.* Currently, we have not identified transcription factors that regulate quercetin biosynthesis in *E. maculata*. Therefore, further identification the related transcription factors and investigation into the mechanism of their action are warranted.

Quercetin is the sole indicator component of *E. maculata* in the Chinese Pharmacopoeia (2020 edition). It determines the quality of the species, and its content varies in various tissues at different stages. However, the molecular mechanism of quercetin biosynthesis that causes the differences in quercetin content in *E. maculata* remains largely unexplored. In the previous study, we collected various tissues (root, stem, leaf, and fruit) of *E. maculata* at different stages and detected quantitative differences in quercetin between the samples. It was found that quercetin content in the same tissue showed a downward trend as *E. maculata* matured^14^, similar to observations during the flower development in *Lonicera macranthoides*^43^. The expression levels of genes related to the biosynthesis of active ingredients can affect their accumulation^44^. For example, the up-regulation of *PAL* and *4CL* expression has been shown to increase the content of flavonoids in *Pyrus bretschneideri* cv. Pingguoli^45^, while overexpression of *CHS* and *CHI* can increase quercetin content in transgenic *Linum usitatissimum*^46^. Conversely, down-regulation of *F3H*, *FLS* and *CHS* expression can reduce rutin content in *Solanum lycopersicum*^47^. To explore the reasons for differences in quercetin content, we revealed key DEGs related to quercetin biosynthesis in the same tissues at different stages. In the same tissues, *PAL*, *4CL*, *CHS*, *CHI*, and *FLS* showed significantly lower expression at the reproductive stage compared to the vegetative stage. In the previous studies^14,48^, *CHS* and *FLS* were selected for RT-qPCR validation. The expression levels of these two genes in the same tissue showed a down-regulated trend as *E. maculata* matured. Comparative analysis of the selected genes showed a similar expression pattern in RT-qPCR analysis as observed in RNAseq data, suggesting the reliability of the results. The down-regulated trends of *CHS* and *FLS* were basically consistent with the decreasing trend of quercetin accumulation as *E. maculata* matured. These results may imply that as *E. maculata* matured, the decrease in quercetin accumulation is associated the down-regulated expression of key genes in the same tissue. The findings provide a basis for further investigating the mechanism of quercetin biosynthesis, and the cultivation of *E. maculata* varieties with high quercetin content.

## Conclusion

In this study, an investigation was conducted through transcriptome sequencing in various tissues of *E. maculata* at different stages, resulting in a wealth of genetic information and gene expression characteristics. A set of 59 candidate genes associated with quercetin biosynthesis pathway of *E. maculata* were identified, including 17 *PAL*, 3 *C4H*, 16 *4CL*, 5 *CHS*, 4 *CHI*, 1 *F3H*, 4* F3*′*H*, and 9 *FLS.* In the same tissues of *E. maculata*, *PAL*, *4CL*, *CHS*, *CHI*, and *FLS* showed significantly lower expression at the reproductive stage compared to the vegetative stage. These findings not only provide the foundation for further research on the molecular mechanis of quercetin biosynthesis in *E. maculata*, but also provide valuable genetic resources for further research on the biosynthesis pathway of secondary metabolites and biodiversity in *E. maculata* as well as other related species.

## Data Availability

Transcriptome sequence data was submited to the NCBI database (SRA accession number SRP392262, https://www.ncbi.nlm.nih.gov/sra/?term=SRP392262).

## Declarations

### Accordance statement

This research is in compliance with the ‘Convention on International Trade in Endangered Species of Wild Fauna and Flora’ and the ‘IUCN Policy Statement on Research Involving Species at Risk of Extinction’. Any experimental research or field studies involving plants, including the collection of plant material, must adhere to all relevant institutional, national, and international guidelines and legislation.
